# Supplementary material for: Breast cancer cell-derived extracellular vesicles accelerate collagen fibrillogenesis and integrate into the matrix
Source: Mater Today Bio. 2026 Apr 17;38:103133. doi: 10.1016/j.mtbio.2026.103133 (PMC13138046; doi:10.1016/j.mtbio.2026.103133)
Supplement: Multimedia component 1 [file mmc1.pdf]

Breast cancer cell-derived extracellular vesicles accelerate collagen fibrillogenesis and integrate into the matrix

Supplemental Information

Nicky W. Tam*[1], Rumiana Dimova*[1], and Amaia Cipitria*[1, 2, 3]
[1]Max Planck Institute of Colloids and Interfaces, Science Park Golm, 14476 Potsdam, Germany; [2]Group of Bioengineering in Regeneration and Cancer, Biogipuzkoa Health Research Institute, 20014 San Sebastián, Spain; [3]IKERBASQUE, Basque Foundation for Science, 48009 Bilbao, Spain.

* Amaia Cipitria, Rumiana Dimova, and Nicky W. Tam
Email: Amaia.cipitria@mpikg.mpg.de; Amaia.CipitriaSagardia@bio-gipuzkoa.eus, Rumiana.Dimova@mpikg.mpg.de; Nicky.Tam@mpikg.mpg.de

Other supporting materials
All necessary raw and processed data for reproducing our findings, as well as MATLAB and Python scripts used for our analyses can be found in the publicly accessible Edmond repository of the Max Planck Society (https://doi.org/10.17617/3.AMI3GV).

Acronyms used in text	
DLS	Dynamic Light Scattering
DMEM	Dulbecco's Modified Eagle's Medium
DMSO	Dimethyl Sulfoxide
DOPC	1,2-dioleoyl-sn-glycero-3-phosphocholine
DiI	1,1'-Dilinoleyl-3,3,3',3'-Tetramethylindocarbocyanine, 4-Chlorobenzenesulfonate
ECM	Extracellular Matrix
EV	Extracellular Vesicle
FBS	Fetal Bovine Serum
GPMV	Giant Plasma Membrane Vesicle
HBS	HEPES-Buffered Saline
HBS+Ca	Calcium-containing HEPES-Buffered Saline
HEPES	4-(2-hydroxyethyl)-1-piperazineethanesulfonic acid
ITGB1	Integrin β1
LPMV	Large Plasma Membrane Vesicle
LUV	Large Unilamellar Vesicle
MMP	Matrix Metalloproteinase
NEM	N-Ethylmaleimide
PBS	Phosphate-Buffered Saline
RGD	Arginylglycylaspartic Acid
SEC	Size-Exclusion Chromatography
SDS-PAGE	Sodium Dodecyl Sulfate-Polyacrylamide Gel Electrophoresis
tEV	Trypsinized Extracellular Vesicle

Table 1, Acronyms used in the manuscript, in alphabetical order.

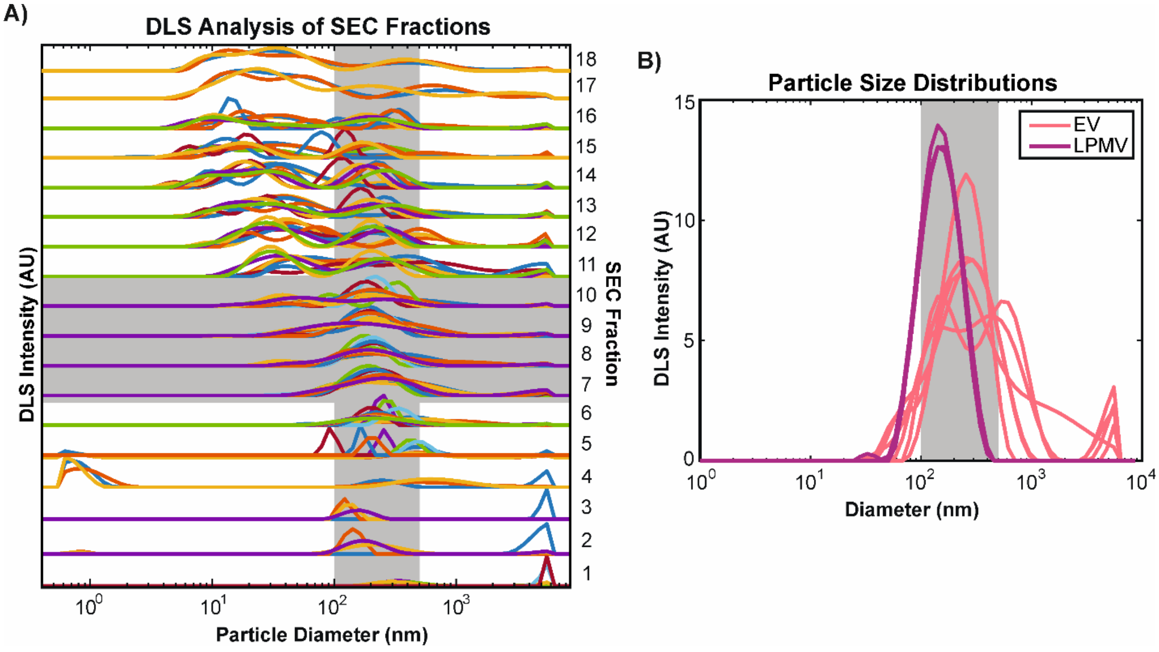

Fig. S1. DLS analysis of EV and LPMV size distribution. Data and figure adapted from the supplemental materials of a previous publication analyzing the same particles (1). A) Size distributions of particles detected by DLS in the different fractions collected during SEC purification of EVs. Different colours represent different replicates. The target particle size range of 100-400nm and the collected fractions are shaded in grey to show that they intersect. Y-axes of the size distributions represent DLS intensity and have been normalized to show relative enrichment as opposed to absolute abundance. B) Comparison of EV (pink) and LPMV (purple) size distributions. EV traces represent pooled EV fractions. EVs are expected to be more polydisperse because LPMVs are extruded with a defined filter pore size. Different curves represent different replicates.

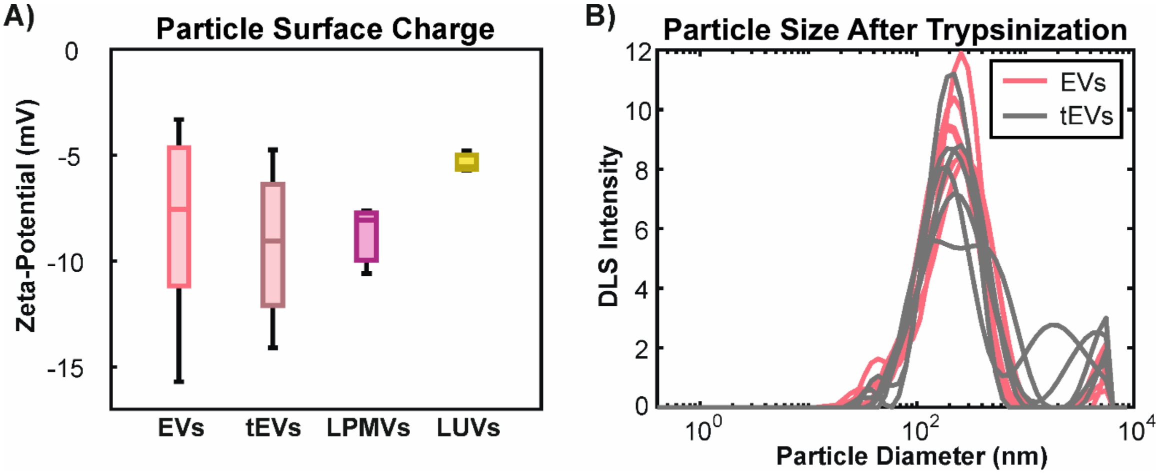

Fig. S2. Effect of trypsinization on EV surface charge and size distribution. Data and figures adapted from the supplemental materials of a previous publication analyzing the same particles (1). A) Relative surface charges of different particles, as represented by zeta-potential measurements in high ionic strength buffers (HBS). EVs and LPMVs have similar surface charge and trypsinization does not appear to significantly affect EV surface charge. Synthetic DOPC LUVs have a slightly less negative charge. B) Size distributions of EVs before (pink) and after (grey) trypsinization. While overall size does not appear to change much, there appears to be slightly more variability in size and the possible existence of aggregates in tEVs (seen here as extra peaks).

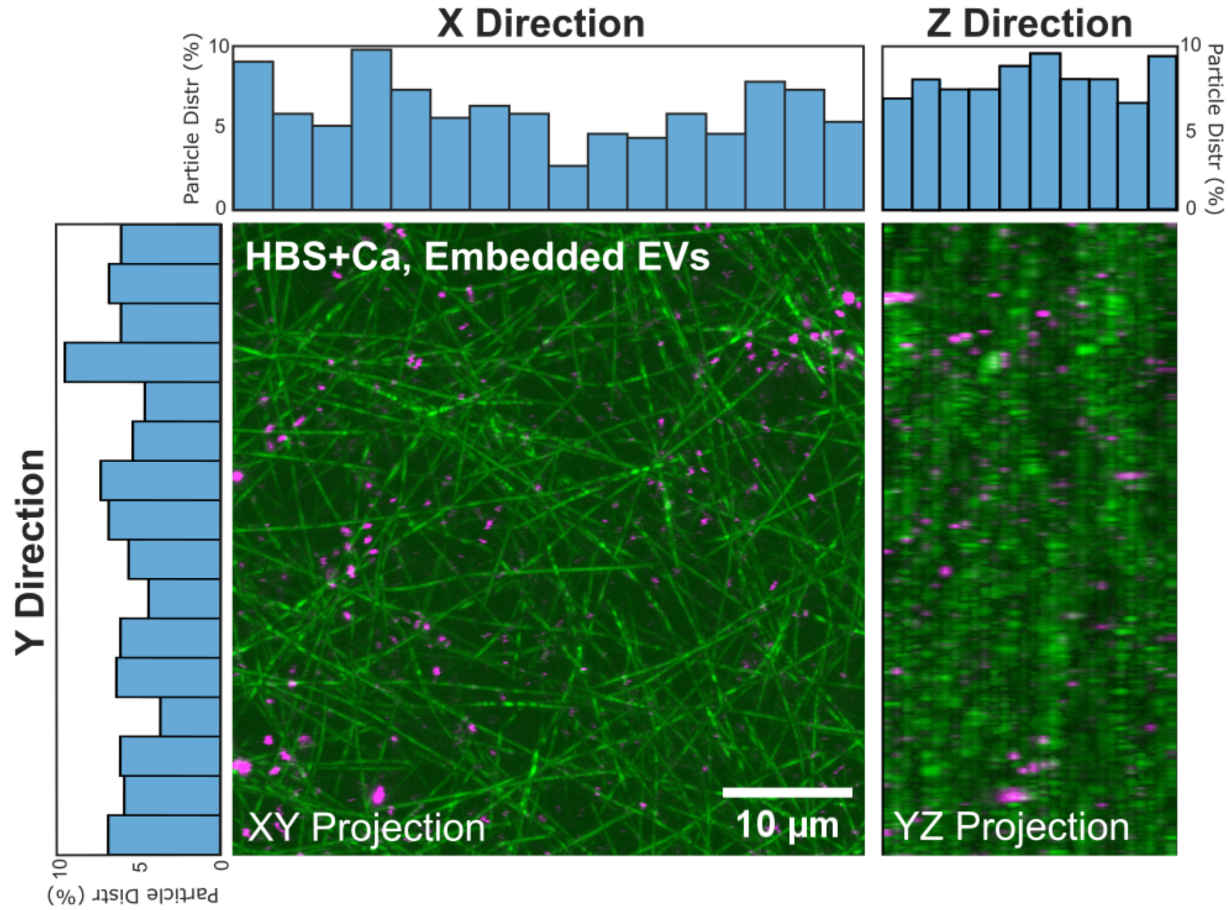

Fig. S3. Particle distributions in collagen I hydrogels formed in the presence of EVs. Projections of the sample on the XY plane and YZ plane are shown with histograms showing the normalized abundance of particles throughout the sample in three dimensions. All three histograms approximate rectangular (uniform) distributions. Some particles are mobile and appear as multiples in images due to being detected by confocal scanning multiple times as they diffuse in the sample. Projections were obtained from stacks of 30 images with a Z-spacing of 0.75 µm (22.5 µm total).

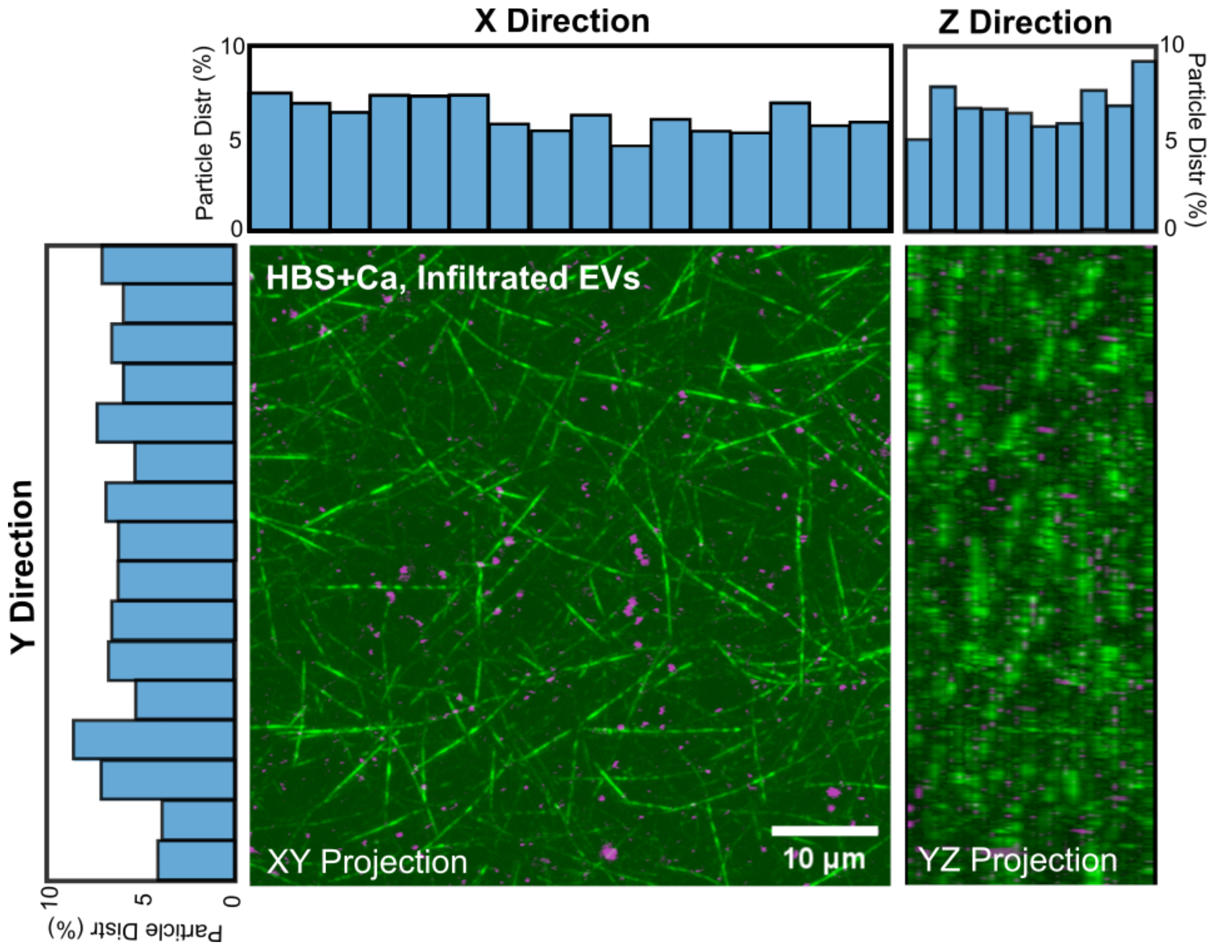

Fig. S4. Distributions of infiltrated particles in pre-formed collagen I hydrogels. Projections on the XY and YZ planes are shown with histograms showing the normalized abundance of particles throughout the sample in three dimensions. All three histograms approximate rectangular (uniform) distributions. Most particles are mobile and therefore appear multiple times in images due to their being detected by the confocal scanning as they diffuse through the sample. Projections were obtained from stacks of 30 images with a Z-spacing of 0.75 μm (22.5 μm total).

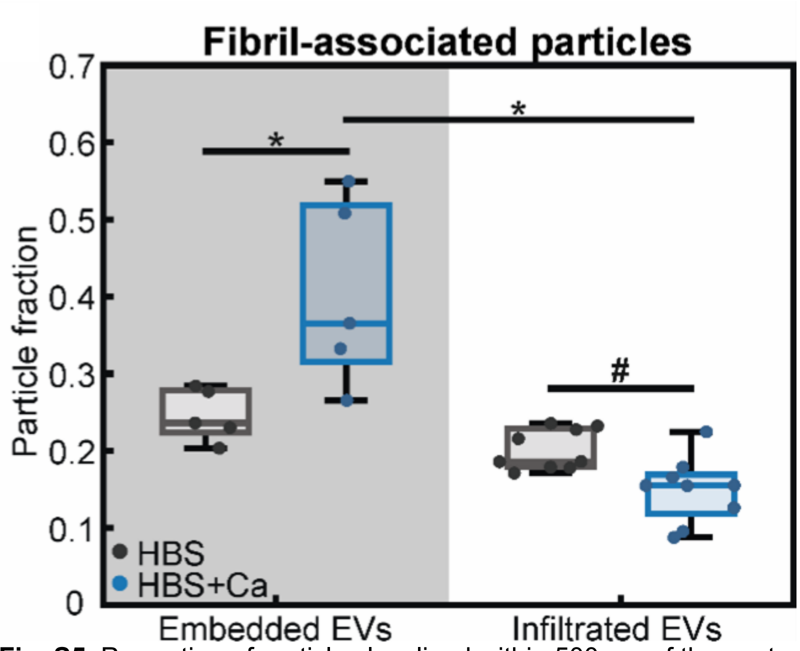

Fig. S5. Proportion of particles localized within 500 nm of the central axis of a collagen fibril. EVs present during gelation of collagen hydrogels supplemented with calcium (Embedded, HBS+Ca) are found at a greater frequency in close proximity to collagen fibrils compared to EVs present with calcium absent (Embedded, HBS) and EVs diffusing into pre-formed collagen hydrogels (Infiltrated). Two-way ANOVA showed statistically significant effects between embedded and infiltrated EVs, as well a significant interaction between this and the presence of calcium. Pair-wise Tukey-Kramer post-hoc analysis for multiple comparisons shows significant differences, as depicted with * ($p<0.01$). A separate two-sample t-test found a significant difference between isolated data, as shown with # ($p<0.01$).

SI References

1. N. W. Tam, A. Becker, A. Mangiarotti, A. Cipitria, R. Dimova, Extracellular Vesicle Mobility in Collagen I Hydrogels Is Influenced by Matrix-Binding Integrins. ACS Nano 18, 29585–29601 (2024).
